# A retrospective clinical investigation for the effectiveness of closed reduction on nasal bone fracture

**DOI:** 10.1186/s40902-019-0236-y

**Published:** 2019-11-27

**Authors:** Byung-Hun Kang, Hyo-Sun Kang, Jeong Joon Han, Seunggon Jung, Hong-Ju Park, Hee-Kyun Oh, Min-Suk Kook

**Affiliations:** Department of Oral and Maxillofacial Surgery, School of Dentistry, Dental Science Research Institute, Chonnam National University, 42, Jebong-ro, Dong-gu, Gwangju, 61469 Republic of Korea

**Keywords:** Nasal bone fracture, Etiology, Closed reduction, Complication

## Abstract

**Background:**

The nasal bone is the most protruding bony structure of the facial bones. Nasal bone fracture is the most common facial bone fracture. The high rate of incidence of nasal bone fracture emphasizes the need for systematical investigation of epidemiology, surgical techniques, and complications after surgery. The objective of this study is to investigate the current trends in the treatment of nasal bone fractures and the effectiveness of closed reduction depending on the severity of the nasal bone fracture.

**Patients and methods:**

A total of 179 patients with a nasal bone fracture from 2009 to 2017 were enrolled. Their clinical examination, patient’s records, and radiographic images of nasal bone fractures were evaluated.

**Results:**

Patients ranged from children to elderly. There were 156 (87.2%) males and 23 (12.8%) females. Traffic accident (36.9%) was the most common cause of nasal fracture. Orbit fracture (44 patients, 24.6%) was the most common fracture associated with a nasal bone fracture.

Complications after surgery included postoperative deformity in 20 (11.2%) patients, nasal obstruction in 11 (6.1%) patients, and olfactory disturbances in 2 (1.1%) patients and patients with more severe nasal bone fractures had higher rates of these complications.

**Conclusion:**

Closed reduction could be performed successfully within 2 weeks after injury.

## Background

The nasal bone is the most protruding bony structure of facial bones, making it susceptible to impact. Thus, nasal bone fracture is the most common facial bone fracture, accounting for about 40% of all facial fractures. It is the third most common fracture of all bone fractures. This is due to the fact that, in addition to being the most protruding facial bone, it is composed of thin membranous bone and therefore has low breaking stress [1].

Although there is debate over what the optimal treatment is for nasal bone fractures [2], Hwang et al. [3] have stated that noninvasive reduction techniques can be used to treat fractures of the nasal bone.

Many studies have shown that the level of satisfaction in patients after surgery is lower for nasal bone fractures compared with that for fractures of other facial bones [4, 5]. In addition, the high rate of incidence of nasal bone fracture emphasizes the need for systematical investigation of epidemiology, surgical techniques, complications after surgery, and so on regarding this fracture. Although there is an abundant amount of research on the demographic data, cause of injury, types of nasal bone fractures, and associated fractures, more research on the types and rate of complications depending on the types or severity of nasal bone fractures is needed.

This study provides a categorization of nasal bone fractures and statistical analysis based on the investigation of surgical techniques, associated fractures, and complications after surgery. Medical records and computed tomography (CT) of 179 patients treated for nasal bone structures in the department of Oral and Maxillofacial Surgery in Chonnam National University Hospital in the past recent 9 years spanning from January of 2009 to December of 2017 were reviewed. Based on this study, the current trends in the treatment of nasal bone fractures and the effectiveness of closed reduction techniques depending on the severity of nasal bone fracture can be evaluated.

## Methods

Subjects of this study were 179 patients diagnosed and treated for nasal bone fracture in the Department of Oral and Maxillofacial Surgery in Chonnam National University Hospital from January of 2009 to December of 2017. Ethical approval was obtained from the Chonnam National University Dental Hospital Institutional Review Board (IRB CNUDH-2019-010).

**Fig. 1:**
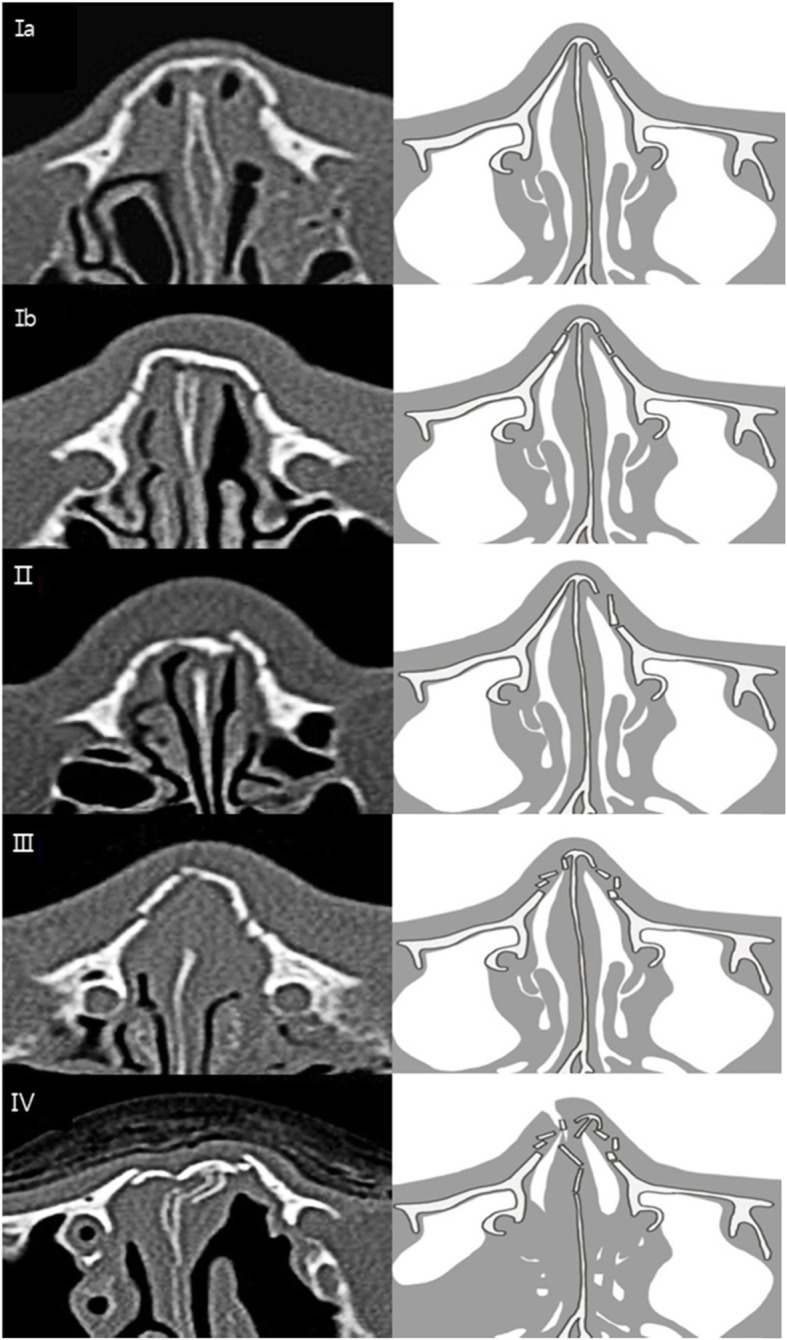
Classification of nasal bone fracture. **Ia** Simple unilateral, non-displaced. **Ib** Simple bilateral, non-displaced. **II** Simple displaced. **III** Closed comminuted. **IV**, Open comminuted or complicated*

Ages, gender, cause of injury, treatment method, associated fractures, and complications were investigated through medical records. CT images were also analyzed. Fractures were classified into the following five categories according to Higuera et al. [6] (Fig. 1, Table 1).

**Table 1:** Classification of nasal bone fracture

Ia	Simple unilateral, non-displaced	
Ib	Simple bilateral, non-displaced	
II	Simple displaced	
III	Closed comminuted	
IV	Open comminuted	
	Any of the above types with	Airway obstruction
		Septal hematoma
		Crush injury
		Severe displacement
	NOE fracture	

## Results

### Gender and age of patients

Average age of patients was 38.2 years old (range, 5~80 years old). Males had about 6.8 times more incidences than females. Out of 179 patients, 156 (87.2%) were males and 23 (12.8%) were females.

Regarding the number of incidences of nasal fractures by age groups, there were 36 patients (20.1%) aged 10–19 years old, which was the highest rate of occurrence among all age groups, followed by 35 patients (19.6%) aged 10–19 years old, 31 patients (17.3%) aged 40–49 years old, 26 patients (14.5%) aged 30–39 years old, 25 patients (14.0%) aged 50–59 years old, 24 patients aged 60 years old and above, and 2 patients (1.1%) aged under 10 years old (Tables 2 and 3).

**Table 2:** Distribution according to age and gender

Age	Male	Female	Number of patients (%)
< 10	1	1	2 (1.1)
10–19	30	5	35 (19.6)
20–29	32	4	36 (20.1)
30–39	25	1	26 (14.5)
40–49	24	7	31 (17.3)
50–59	23	2	25 (14.0)
≥ 60	21	3	24 (13.4)
Total	156 (87.2)	23 (12.8)	179 (100)

**Table 3:** Cause of injury according to age

Age	Fall down	Traffic accidents	Violence	Sports-related trauma	Industrial trauma	Others	Total
< 10	1	1	0	0	0	0	2
10–19	4	11	16	1	0	3	35
20–29	9	15	8	1	1	2	36
30–39	8	6	9	2	1	0	26
40–49	6	8	11	1	4	1	31
50–59	10	10	3	0	1	1	25
≥ 60	6	15	2	0	1	0	24
Total	44	66	49	5	8	7	179

### Cause of nasal fractures

In the current study, the most common cause of nasal fractures was traffic accident (66 cases, 36.9%), followed by violence (49 cases, 27.4%), falling down (44 cases, 24.6%), industrial accidents (8 cases, 4.5%), other incidences (7 cases, 3.9%), and sports-related trauma (5 cases, 2.8%).

### Reduction period and types

Reduction of the nasal bone was performed at an average of 7.7 days (range, 0~33 days) after fracture. Closed reduction was performed for 139 (77.7%) patients, which was the most. Open reduction was performed for 9 (5.0%) patients. Recovery of injury was observed without any type of reduction in 31 (17.3%) patients (Table 4).

**Table 4:** Types of nasal bone fracture and treatment

Fracture classification	Open reduction	Closed reduction	Observation	Total
Ia	0	5	8	13
Ib	0	1	2	3
II	4	56	15	75
III	0	28	4	32
IV	5	49	2	56
Total	9	139	31	179

### Types of nasal bone fracture and its treatment

There were 13 (7.3%) patients with type Ia fracture, 3 (1.7%) patients with type Ib fracture, 75 (41.9%) patients with type II fracture, 32 (17.9%) patients with type III fracture, and 56 (31.3%) patients with type IV fracture (Table 4).

For type Ia fractures, closed reduction was performed for 38.5% (*n* = 5) of cases while only observation was carried out for 61.5% (*n* = 8) of cases. For type Ib fractures, closed reduction was performed for 33.3% (*n* = 1) of cases while only observation was carried out for 66.7% (*n* = 2) of cases. For type II fractures, 5.3% (*n* = 4) of cases were treated with open reduction and 74.7% (*n* = 56) of cases were treated with closed reduction while only observation was carried out for 20.0% (*n* = 15) of cases. For type III fractures, 87.5% (*n* = 28) of cases were treated with closed reduction while only observation was carried out for 12.5% (*n* = 4) of cases. Finally, for type IV fractures, 8.9% (*n* = 5) of cases were treated with open reduction and 87.5% (*n* = 49) of cases were treated with closed reduction while only observation was carried out for 3.6% (*n* = 2) of cases.

### Fractures associated with nasal bone fracture

Regarding associated fractures, 17 (9.5%) patients with Le Fort I fracture, 6 (3.4%) patients with Le Fort II fracture, 2 (1.1%) patients with Le Fort III fracture, 4 (2.2%) patients with NOE fracture, 33 (18.4%) patients with ZMC fracture, 18 (10.1%) patients with maxillary fracture, 44 (24.6%) patients with orbital fracture, 10 (5.6%) patients with frontal bone fracture, and 10 (5.6%) patients with alveolar bone fracture were found. Duplications in counting for associated fractures were allowed (Table 5).

**Table 5:** Types of associated fracture

Associated fractures	Ia	Ib	II	III	IV	Total
None	7	2	39	18	23	89
Le Fort I	2	0	4	4	7	17
Le Fort II	1	0	0	1	4	6
Le Fort III	1	0	0	0	1	2
NOE fx	0	0	0	0	4	4
ZMC fx	3	0	17	6	7	33
Maxillary fx	0	0	10	2	6	18
Orbital fx	0	1	23	5	15	44
Frontal fx	2	0	3	1	4	10
Alveolar fx	0	0	2	1	7	10
Total	16	3	98	38	78	233

### Complications

Complications after treatment of nasal bone fracture included 11 (6.1%) patients with nasal obstruction, 20 (11.2%) patients with postoperative deformity, and 2 (1.1%) patients with olfactory disturbances and patients with more severe nasal bone fractures had higher rates of these complications (Table 6).

**Table 6:** Types of complications

Fracture classification	None	Nasal obstruction	Postoperative deformity	Olfactory disturbances	Total
Ia	12	1	0	0	13
Ib	3	0	0	0	3
II	71	1	2	1	75
III	24	1	7	0	32
IV	39	8	11	1	59
Total	149	11	20	2	182

## Discussion

Part of the body that plays the most important role in our ability to distinguish individuals is the face, and the nose is the most noticeable part of the face [7]. The nose is also the most protruding structure. Therefore, it is very susceptible to injury. In addition, even a small deformity in the nasal bone or cartilage is very noticeable as it affects the overall esthetics of the face. Furthermore, nasal bone fracture is the most common fracture of the face. Its incidence rate has increased due to changes in lifestyle and increases in traffic accidents, thus creating a need for an epidemiological study [8]. Regarding the frequency of nasal bone fractures by gender, the ratio of male to female has been analyzed by Marco et al. [9] to be 4.1:1. Turvey et al. [10] have reported a ratio of 3:1, and Nishioka et al. [11] have shown a ratio of 2.3:1. These studies all indicate a higher frequency in males. In the present study, there was a significantly higher frequency of nasal bone fracture in males with a ratio of 6.8:1. Regarding age groups, Hwang et al. [3] have observed that nasal bone fractures occur most commonly in patients in their 20s (31.8%), followed by patients in their teens, 30s and 40s (22.3%, 19.7%, and 16.1% respectively). Oh et al. [8] have also observed that nasal bone fractures occur most commonly in patients in their 20s (31.7%), followed by patients in their teens (22.6%), 30s (20.0%), and 40s (16.1%).

Most research studies on incident rates of nasal bone fractures according to age groups have shown that the highest rate of incidence occurs in males aged 15 to 40 years old. In the present study, 36 (20.1%) patients aged 20–29 years old, which had the highest rate of occurrence among age groups, followed by 35 (19.6%) patients aged 10–19 years old, 31 (17.3%) patients aged 40–49 years old, 26 (14.5%) patients aged 30–39 years old, 25 (14.0%) patients aged 50–59 years old, 24 (13.4%) patients aged 60 years old and above, and 2 (1.1%) patients aged under 10 years old. Similar to most researches, males aged 10 to 40 years old had the highest rate of nasal bone fracture in the present study. The reason for this can be attributed to the fact that most males in this age group take part in labor, physical activity, violence, and so on.

Causes of nasal bone fracture are known to differ by age and region. Hwang et al. [12] have reported that the most common causes of nasal bone fracture in adults are fights (36.3%), traffic accidents (20.8%), sports (15.3%), and falls (13.4%) while the most common causes in children are sports (59.3%), fights (10.8%), traffic accidents (8.3%), collisions (5.0%), and falls (3.3%). Causes of nasal bone fractures also vary by region. Fighting is the most common cause in Asia (36.7%), South America (46.5%), and Europe (40.8%) while traffic accident is the most common cause in North America (33.6%). In the current study, the most common cause of nasal fractures was traffic accident (66 cases, 36.9%), followed by violence (49 cases, 27.4%), falling down (44 cases, 24.6%), industrial accidents (8 cases, 4.5%), other incidences (7 cases, 3.9%), and sports-related trauma (5 cases, 2.8%).

Regarding fractures associated with nasal bone fractures, Yang et al. [13] have reported that maxillary fracture is the most common fracture (50%), followed by mandible fracture (20%) and zygomatic bone fracture (15%). In the present study, 17 (9.5%) patients incurred Le Fort I fracture, 6 (3.4%) patients incurred Le Fort II fracture, 2 (1.1%) patients incurred Le Fort III fracture, 4 (2.2%) patients incurred NOE fracture, 33 (18.4%) patients incurred ZMC fracture, 18 (10.1%) patients incurred maxillary fracture, 44 (24.6%) patients incurred orbital fracture, 10 (5.6%) patients incurred frontal bone fracture, and 10 (5.6%) patients incurred alveolar bone fracture. The reason for such results might be because causes of fractures such as traffic accidents, falling down, violence, and so on often lead to stress to the middle portion of the face because the nasal bone is the most protruding structure. Thus, bones closest to the nasal bone have higher rates of associated fractures.

Although most nasal bone fractures can be treated with closed reduction, there is a difference in opinion about what the appropriate time for reduction is depending on the doctor [14]. Rohrich et al. [14] have stated that reduction should be carried out within 7 days of the fracture while it should be carried out within 10 days for adults. However, Harrison [4] claims that 3–7 days for children and 5–10 days for adults would be the appropriate time for reduction. Goode and Spooner [15] have suggested that the appropriate time for reduction is 2–3 days after fracture when edema disappears. Han [16] claims that in the case of nasal bone fractures with multiple fracture segments, reduction after 2 weeks can result in the best outcome. In the present study, reduction was carried out in an average of 7.7 days (range, 0 to 33 days) after the fracture. Reduction was not carried out for minor fractures that had no effect on the appearance of the face. In these instances, precise evaluation of facial appearance is needed. Therefore, it is advantageous to decide whether or not to carry out surgical procedures after the edema has dissipated after injury.

Regarding complications after the reduction of nasal bone fractures, Hwang et al. [17] reported that nasal deformity occurred in 10.4% of patients. In addition, 10.0% suffered septal deviation, 10.5% suffered nasal obstruction, 3.1% suffered epiphora, 3.1% suffered diplopia, and 37.7% of patients suffered olfactory disturbances. In the present study, 11 (6.1%) patients suffered nasal obstruction, 20 (11.2%) patients suffered postoperative deformity, and 2 (1.1%) patients suffered olfactory disturbances. Anatomically, olfactory epithelial cells are scattered on the superior part of the nasal cavity and bilaterally between the septum and the medial portion of the superior nasal concha [18]. Also, olfactory epithelium might be present above the middle turbinate superiorly and below the cribriform plate inferiorly. The reason for high rates of olfactory disturbances after the reduction of nasal bone fractures is that olfactory epithelial cells located on superior nasal concha or supreme nasal turbinate can get damaged during a procedure [19]. In the present study, olfactory disturbance was observed in only 2 patients. Thus, it could be inferred that closed reduction was carried out delicately.

In the current study, fractures were classified into five categories as stated by Higuera et al. [6]*.* This method of categorization does not take displacement into consideration. Rather, it uses subjective judgment. Therefore, it has a clear limitation as an objective method of categorization.

## Conclusion

According to data of this study, nasal bone fractures occurred at similar rates in all age groups and predominantly in males. Successful closed reduction could be performed within 2 weeks after injury.

## Data Availability

All data analyzed during this study are included in this published article.

## Abbreviations

ZMC: Zygomaticomaxillary complex
NOE: Naso-orbito-ethmoid
CT: Computed tomography
